# Interventional Cardio-Oncology: Adding a New Dimension to the Cardio-Oncology Field

**DOI:** 10.3389/fcvm.2018.00048

**Published:** 2018-05-17

**Authors:** Victor Y. Liu, Ali M. Agha, Juan Lopez-Mattei, Nicolas Palaskas, Peter Kim, Kara Thompson, Elie Mouhayar, Konstantinos Marmagkiolis, Saamir A. Hassan, Kaveh Karimzad, Cezar A. Iliescu

**Affiliations:** ^1^Department of Internal Medicine, McGovern Medical School at The University of Texas Health Science Center at Houston, Houston, TX, United States; ^2^Department of Cardiology, Division of Internal Medicine, The University of Texas MD Anderson Cancer Center, Houston, TX, United States; ^3^Department of Diagnostic Radiology, Division of Diagnostic Imaging, The University of Texas MD Anderson Cancer Center, Houston, TX, United States; ^4^Florida Hospital Pepin Heart Institute, Tampa, FL, United States

**Keywords:** interventional cardio-oncology, optical coherence tomography, intravascular imaging, thrombocytopenia, fractional flow reserve, instantaneous free-wave ratio, transcatheter aortic valve replacement, takotsubo cardiomyopathy

## Abstract

The management of cardiovascular disease in patients with active cancer presents a unique challenge in interventional cardiology. Cancer patients often suffer from significant comorbidities such as thrombocytopenia and coagulopathic and/or hypercoagulable states, which complicates invasive evaluation and can specifically be associated with an increased risk for vascular access complications. Furthermore, anticancer therapies cause injury to the vascular endothelium as well as the myocardium. Meanwhile, improvements in diagnosis and treatment of various cancers have contributed to an increase in overall survival rates in cancer patients. Proper management of this patient population is unclear, as cancer patients are largely excluded from randomized clinical trials on percutaneous coronary intervention (PCI) and national PCI registries. In this review, we will discuss the role of different safety measures that can be applied prior to and during these invasive cardiovascular procedures as well as the role of intravascular imaging techniques in managing these high risk patients.

## Interventional Cardio-Oncology

There are approximately 14.5 million cancer survivors in the United States alone, a number that is expected to reach 20 million within the next ten years. It is estimated that in 2016, over 1.6 million new cases of cancer were diagnosed in the United States, and approximately 600,000 people died from the disease (1). However, the death rate from cancer in the United States continues to decline. With improvements in early diagnosis, monitoring and treatment of various malignancies, cancer patients often are at higher risk of mortality from cardiovascular disease rather than recurrence of their cancer.

Patients with malignancies present challenges to the management of cardiovascular disease. They are often frail, particularly those with advanced age and those who sustain off-target effects from cancer treatments. Comorbidities must be appropriately evaluated and addressed for optimal management of cardiovascular health. Furthermore, cancer patients often have significant time constraints placed upon them, and care must be coordinated around various diagnostic and/or therapeutic oncologic procedures. Outside of the acute setting, cancer survivors experience increased cardiovascular morbidity and mortality across all age groups (2, 3). Cancer is intrinsically linked to heart disease via multiple mechanisms: common risk factors, chronic inflammatory states brought on by malignancies, and cardiac and vascular toxicities of cancer therapy.

### Coronary Artery Disease (CAD)

Cardiovascular disease and cancer share similar risk factors: increasing age, cigarette smoking, obesity, diabetes, hypertension, hyperlipidemia, and physical inactivity (4). These risk factors increase the short- and long-term cardiac mortality in cancer patients (5). At the time of cancer diagnosis there is a high prevalence of cardiac disease, which is often exacerbated by necessary cancer therapeutic agents (6). The pathogenesis and progression of cancer and cardiovascular disease also share an increased state of inflammation. Inflammation and endothelial damage are key in the development of atherosclerosis and plaque thrombosis (7, 8). Meanwhile, cancerous cells produce pro-inflammatory cytokines and chemokines that damage the endothelium and increase permeability of the microvasculature (9). This allows the formation of plaques as low-density lipoprotein cholesterol particles invade damaged vascular intima, creating a pro-atherosclerotic effect and contributing to increased risk of CAD in cancer patients.

Cancer therapeutic agents frequently cause cardiovascular complications, due to direct toxicity to the vascular endothelium and myocardium. Complications may include anginal chest pain, hypertension, acute coronary syndromes (ACS), stroke, arterial thrombosis leading to limb ischemia, venous thrombosis, arrhythmias, and heart failure. Development of cardiotoxic side effects from chemotherapy depends on several factors including the choice of drug, dose of drug administered, interval of administration, cumulative dose, route of administration, and association with radiotherapy (10). Those at the extremes of age are at increased risk of cardio-toxicity from chemotherapeutic agents, as are those with preexisting cardiovascular risk factors/disease and those with a history of radiation therapy to the chest (11).

Several forms of antineoplastic therapy including chemotherapy, hormonal therapy, and radiotherapy have been associated with a higher risk of atherosclerosis, CAD and cardiac ischemia (12). The presumed mechanism of cancer therapy causing CAD is highly variable and often unknown. Coronary vasospasm is one of the most frequently noted adverse effects of cancer therapy that may lead to myocardial ischemia or infarction, as seen in agents such as sorafenib, 5-fluorouracil and capecitabine (13–15). Apoptosis of endothelial cells, resulting in vasospastic angina and myocardial infarction, is an adverse effect related to antineoplastic agents such as etoposide, bleomycin, bevacizumab and vinblastine (16). Bevacizumab specifically, by inhibiting vascular endothelial growth factor (VEGF), increases expression of proinflammatory genes and decreases endothelial cell production of the vasodilator nitric oxide, causing increased platelet activity (17, 18). One study found that addition of bevacizumab to chemotherapy resulted in increased incidence of arterial thromboembolic events, from 3.1 events in those with standard chemotherapy compared to 5.5 events per 100 person-years in those additionally treated with bevacizumab (19). A recent meta-analysis found that bevacizumab increased the risk of arterial adverse events, including cardiac and cerebral ischemia, venous thromboses/pulmonary emboli, bleeding, and hypertension with higher risks in patients taking higher doses (18). Lenalidomide is an immunomodulatory agent used in the management of multiple myeloma that similarly increases the risk for both arterial and venous thrombo-embolic events, including myocardial infarction and cerebrovascular accidents (20).

Accelerated progression of atherosclerosis and elevated risk of ischemic events has similarly been reported in cancer survivors on long-term hormone deprivation therapies such as gonadotropin-releasing hormone agonists (21–24) or aromatase inhibitors (anastrozole, letrozole, exemestane) (25). Tamoxifen is a selective estrogen receptor modulator that does not seem to have increased cardiovascular risk based on randomized, placebo-controlled trials (26), but does increase thromboembolic risk (25).

Accelerated atherosclerosis has been well documented in cancer patients with exposure to radiotherapy. These survivors experience higher risk of ischemic heart disease beginning as soon as 5 years following exposure and continuing for life. The risk of major adverse events is dependent on the radiation dose received, with an increased risk of 7.4% per gray of radiation. There is not a safe threshold of exposure below which there is zero risk (27). Radiation to the heart causes these effects through direct damage to the endothelium resulting in inflammatory changes such as increased monocyte attachment which, in combination with elevated cholesterol, initiates the formation of fatty streaks and ultimately atherosclerosis (28–30). Damage also occurs to the microvasculature, causing inflammation and thrombus formation which obstructs the microvasculature ultimately leading to ischemia, fibrosis, and death of myocardial cells (31, 32). Thus, patients with history of exposure to chemotherapy or cardiac radiation may be considered for screening every 5 years with an ankle-brachial index, carotid ultrasound, stress test, and/or coronary CT angiography for evidence of advancing coronary and peripheral atherosclerosis (33). Table 1 contains a list of the cardiovascular effects associated with several anticancer therapies.

Recently published data on 279,719 pairs of patients with a new primary diagnosis of cancer and matched control patients found a 6 month cumulative incidence of arterial thromboembolism in 4.7% of cancer patients compared with 2.2% in control patients (34). Furthermore, risk for thromboembolism generally resolved by a year following cancer diagnosis and correlated with cancer stage. Since cancer patients are at higher risk for thrombotic events immediately following diagnosis, an additional concern for the cardio-oncologist is to achieve the proper balance of antiplatelet, antithrombotic, and statin medications in patients with potential bleeding diathesis.

### Thrombocytopenia

Thrombocytopenia is frequent in cancer patients, occurring in anywhere from 10 to 25% of solid tumor patients treated with intensive chemotherapy as well as most acute leukemia, lymphoma, myelodysplastic syndrome and multiple myeloma patients (35). Complicating their management, 15 to 25% of thrombocytopenic patients experience thrombocytopenia refractory to platelet transfusion, which is defined as failure to increase platelet counts by 10,000/μl or more after transfusion of an appropriate dose of platelets or >3,000/μl increase per unit (36). Clinical studies suggest that platelet function is more important than platelet count (37). Prophylactic platelet transfusion is not recommended in the general adult inpatient population for platelet counts over 10,000/μl (38). For patients receiving therapy for urologic, gynecologic, colorectal tumors or melanoma, or in extreme cases of known necrotic tumors, transfusion can be considered when the platelet count dips below 20,000/μl (39). Following transfusion, platelet count must be remeasured to ensure the desired level has been reached. In patients undergoing invasive procedures, platelets should be available on short notice in case a bleeding event occurs. Many cancer patients have a long history of receiving transfusions; for alloimmunized patients, histocompatible platelets must be available.

When cancer patients with thrombocytopenia require lifesaving interventions, thromboelastography (TEG) may be utilized in some centers to assess their ability to undergo certain procedures. TEG is a viscoelastic method of blood clotting assessment used at the bedside to analyze the entire process of clotting, including both platelet and coagulation function (40). TEG may determine whether pericardiocentesis would be safe in patients with platelet counts below 30,000 (36), but the current data is limited. Abnormal TEG results require correction prior to the procedure with a platelet transfusion or the necessary blood products. Unfortunately, data is lacking to support the use of TEG in PCI management, presenting a challenge in patients requiring PCI in this setting. As TEG is available at few centers, this limited experience is largely drawn from the anesthesia, cardiovascular and liver transplant surgical literature.

Thrombocytopenia is traditionally considered a relative contraindication to pericardiocentesis for patients with pericardial effusions (41). However, a recent study showed that pericardiocentesis was safe and effective in the setting of malignancy and thrombocytopenia (36, 42). Pericardiocentesis on thrombocytopenic patients may be performed under echocardiographic and/or fluoroscopic guidance. In appropriate patients, an intercostal approach is preferred to avoid possible trauma to the liver from a subxiphoid approach. Care must be taken to ensure that the needle is placed appropriately above the specific rib margin to avoid damage to the intercostal vessels and nerves (42, 43). Micro-puncture and small sheath size are recommended to minimize procedural risks (44).

Due to lower risk of bleeding complications such as retroperitoneal hemorrhage, pseudoaneurysm, arterio-venous fistula and excessive bleeding, radial artery access is preferred for invasive diagnosis and management of CAD in patients with thrombocytopenia (45). These patients still require anticoagulation administration while undergoing transradial diagnostic catheterizations; unfractionated heparin can be given intra-arterially or intravenously at decreased doses of 50 U/kg or 3,000 units in cancer patients platelet counts below 50,000/μl undergoing cardiac catheterization via radial access. Even in thrombocytopenic patients receiving anticoagulation and antiplatelet therapy, reductions in bleeding complications can be achieved. Meanwhile, radial access site catheterization allows for early ambulation, decreasing risk of complications from venous thrombosis (46, 47)

Evidence is lacking on dual antiplatelet therapy (DAPT) for stents placed in the setting of thrombocytopenia. However, given the hypercoagulable state that cancer presents, thrombocytopenic patients who undergo stent placement should receive DAPT. For the general populace, a recent focused update of the ACC/AHA guidelines on duration of DAPT in CAD patients offered a class IIb recommendation that ACS patients treated with DAPT following DES implantation with a high risk of bleeding or severe bleeding complications can be reasonably discontinued after 6 months of P2Y12 therapy (48).

## Takotsubo Cardiomyopathy

Stress-induced cardiomyopathy (SC), also known as Takotsubo cardiomyopathy, mimics the clinical presentation of acute myocardial infarction with symptoms such as chest pain, dyspnea, hypotension, and electrocardiographic changes mimicking STEMI or NSTEMI (49). In patients undergoing cardiac catheterization for ACS there is an approximately 1% incidence of this disorder; the incidence in the cancer population appears to be significantly higher at 10–20% (50). While in the general population SC is appropriately categorized and discussed under the subject of “cardiomyopathy,” this surprising insight suggests that the interventional cardio-oncologist is more likely to encounter SC and should be aware of the high incidence of SC in the cancer population as compared to the general population. In patients with SC, the circulating epinephrine and norepinephrine levels released from chromaffin cells as well as norepinephrine from sympathetic nerve terminals are elevated during the acute course of clinical presentation, suggesting that this cardiomyopathy is driven by excess adrenergic stimulation of cardiomyocytes (51). However, there is no clear explanation for the pathogenesis of this cardiomyopathy, and the mechanisms involved are likely to be heterogeneous and multifactorial. Possible mechanisms include emotional stress when receiving a frightening cancer diagnosis, catecholamine-induced microvascular vasospasm, inadequate increase in cardiac sympathetic nervous activity, modification of cardiomyocyte adrenergic receptors by the underlying malignancy, and reduction in estrogen (52). Antineoplastic agents such as 5-FU, Sunitinib, and Cytarabine may cause SC as an adverse effect (53, 54). Prognosis in patients with SC is generally good in the absence of significant underlying comorbidities. Anticancer therapy can be resumed within 2 to 4 weeks, and beta-blockers should be utilized indefinitely to reduce sympathetic stimulation of the heart.

## Intracoronary Imaging (Optical Coherence Tomography)

Optical Coherence Tomography (OCT) is an important intravascular imaging modality in cardio-oncology. OCT is used for risk stratification of plaques, as plaque architecture affects risk of thrombosis. Those with a thin fibrous cap covering large thrombogenic cores, known as thin-cap fibroatheromas, are more susceptible to rupture and atherothrombosis (55).

A non-cardiac surgical procedure is unpredictably required within 12 months following stent implantation in approximately 5% of patients with drug-eluting stents (DES) (56). Recent guidelines recommend DAPT for only six months with newer generation DES outside a setting of ACS (48). Moreover, a recent data analysis reported that discontinuation of DAPT 3 to 6 months following (predominantly new-generation) DES placement was not associated with an early increase in major adverse cardiac and cerebrovascular events (57). In fact, greater than 12 months of DAPT therapy was associated with an early increase in such events. In patients with newly diagnosed or existing cancer, DAPT may need to be prematurely discontinued for diagnostic biopsies, surgery or initiation of cancer therapy. Furthermore, the risk of thrombosis is increased due to the prothrombotic state of cancer patients (34). The optimal duration of DAPT therapy in cancer patients receiving coronary stents during the periprocedural period is unclear. The management of cancer patients with recent stent placement requiring urgent DAPT discontinuation remains largely empirical.

OCT can be useful in identifying whether a coronary stent has sufficiently healed and whether discontinuation of DAPT may be appropriate for the clinical scenario. Intravascular imaging such as intravenous ultrasound (IVUS) or OCT after stent placement ensures optimal stent expansion and apposition and absence of complications, given the potential for early DAPT interruption. A recently published single-center prospective study in cancer patients with DES within the past 12 months requiring premature DAPT discontinuation outlined a comprehensive strategy for determining the proper method of discontinuing DAPT in cancer patients (58). Patients classified as low risk were considered to have appropriate stent strut coverage, expansion, apposition, and the absence of in-stent restenosis or intraluminal masses. Low risk patients were allowed to temporarily discontinue DAPT and proceed with cancer related procedures. The incidence of adverse cardiovascular events was assessed after the procedure and at 12 months. Of 40 patients in the study, 27 low-risk by OCT criteria temporarily discontinued DAPT. The remaining 13 patients with one or more OCT findings were considered high risk and underwent bridging with low-molecular weight heparin and the appropriate further endovascular treatment. No cardiovascular events occurred in the low risk group, and one myocardial infarction occurred in the high-risk group. There were no cardiovascular deaths, but a total of 14 non-cardiac deaths occurred before 12 months due to cancer progression or cancer therapy. The median time between stent placement and follow-up OCT was 5.2 months (1.1–11.6 months), with 40% of patients having follow-up within 3 months of stenting. The median time interval over which DAPT was discontinued was 6 days (5–36 days), with 38.5% of patients discontinuing DAPT for over 7 days. It has been suggested that the aforementioned vascular toxicity of various antineoplastic agents can cause delayed stent endotheliazation in cancer patients, demonstrating the utility of real-time imaging of coronary stents to determine if stents are appropriately positioned for suspension of DAPT instead of applying broad, potentially inaccurate timelines for DAPT administration and discontinuation.

While further evidence regarding early discontinuation of DAPT is required to establish its safety and efficacy, an OCT-guided strategy is promising to identify cancer patients who have received DES who may need discontinuation of DAPT to proceed with cancer-related surgery and procedures.

### Fractional Flow Reserve (FFR)-Guided PCI

FFR is a well established method of quantifying the functional severity of coronary artery stenosis during coronary angiography. Maximal hyperemia is induced with intravenous adenosine, allowing for correlation between blood flow and the blood pressure within a coronary artery (59). Comparison of distal coronary pressure to mean aortic pressure provides a functional evaluation of several hemodynamic parameters such as the mass of myocardium supplied by a specific coronary vessel, collateral blood flow and myocardial viability. This ratio provided by FFR adds a functional component to the anatomic assessment of lesion severity already provided by conventional coronary angiography.

The DEFER (Percutaneous Coronary Intervention of Functionally Nonsignificant Stenosis) trial showed that PCI can be safely deferred in patients with FFR above 0.75, with similar event-free survival and symptom recurrence between patients who deferred PCI and those who received PCI (60). group which deferred PCI had decreased rates of myocardial infarction at 15 year follow-up versus the group which received an intervention (61). The FAME study showed that routine measurement of FFR during PCI reduced death, myocardial infarction, and repeat revascularization at 1 year. By using a FFR-guided strategy, there was reduced usage of stents without decrease in functional status, quality of life, or procedure length (62). Several additional studies in recent years have confirmed the safety and reliability of FFR guided decision-making regarding PCI.

The American College of Cardiology guidelines on coronary revascularization state that FFR is reasonable to assess intermediate coronary lesions in the 50 to 70% diameter stenosis range, and can be useful for guiding revascularization decisions in CAD patients (Class IIa, Level A) (63). The most recent version of the appropriate use criteria on coronary revascularization endorses FFR for functional lesion assessment in CAD, as well as, the expanded use of intracoronary physiological testing (64).

Instantaneous wave-free ratio (iFR) is a more recent physiological method that assesses the functional severity of coronary stenoses without the need of hyperemic agents, based on the concept that a translesional gradient should be detectable at rest to have a significant effect on the delivery of blood to the myocardium. iFR is measured during the wave-free period of diastole, a portion of the cardiac cycle suitable for a pressure measurement of the hemodynamic impact of coronary stenosis (65). iFR can serve as a method to spare 60 to 70% of patients from administration of adenosine, which can cause dangerous side effects such as bradycardia and atrioventricular block (66), and multiple trials have shown that iFR is non-inferior to FFR in guiding PCI in the absence of ACS with respect to 1 year risk of all-cause mortality, nonfatal MI or unplanned revascularization (67). Additional benefits of iFR over FFR include shorter procedure length, lower incidence of patient-related discomfort, and the ability to assess serial lesions (68).

Data regarding use of FFR- and iFR-guided PCI in cancer patients is lacking. However, given its reliability in assessing the functional importance of coronary artery stenoses, we believe these are essential tools in the evaluation of patients with active malignancies undergoing cardiac evaluation. High-risk cancer patients with multiple comorbidities but nonsignificant stenosis measured by coronary physiology could avoid further invasive diagnostic or unnecessary therapeutic cardiovascular procedures. Given the risks that antiplatelet therapies present in cancer patients and the complexities associated with cancer care, deferral or avoidance of unnecessary stent placements that are associated with their own risks of thrombosis and would require that patients transiently be placed on DAPT is another major benefit, decreasing the risk of perioperative or chemotherapy-related bleeding complications.

### Transcatheter Aortic Valve Replacement (TAVR)

TAVR was initially utilized as a treatment for patients with severe aortic stenosis (AS) and prohibitively high surgical risk (69). Recently, TAVR proved to be a safe and effective alternative to surgical aortic valve replacement (SAVR) in patients at intermediate surgical risk (70, 71). While several newer trials are still underway, there is no data on TAVR in cancer patients and cancer patients have been excluded from most TAVR studies (72). Meanwhile, the presence of cancer remains a common reason for declining surgical intervention in patients with severe aortic stenosis (73). Cancer survivors are at higher risk for SAVR due to prohibitive anatomy (e.g., mediastinal fibrosis, severe lung disease, porcelain aorta, and prior thoracic surgeries or chest radiation). This presents a problem, as cancer patients with severe AS who do receive AVR have improved survival, regardless of their cancer status (74). One case series involving six cancer patients demonstrated that balloon aortic valvuloplasty, which can be used as a bridge to SAVR, TAVR or non-cardiac surgery, is a viable option in cancer patients with severe AS (75). Recent expert consensus suggests that balloon aortic valvuloplasty and TAVR can be used as a palliative measure for symptomatic AS in cancer patients (39). One concern regarding TAVR is the increased rates of subclinical leaflet thrombosis and reduced leaflet motion seen in bioprosthetic aortic valves when compared to SAVR (76–78), especially in cancer patients who may already be hypercoagulable. Increased rates of transient ischemic attacks were associated with subclinical leaflet thrombosis, and therapeutic anticoagulation was found to resolve the condition (76). While the clinical significance of this finding is unclear, TAVR is associated with excellent outcomes which may be further improved with thorough investigation of this complication.

## Conclusion

Interventional cardio-oncology is a new field in search of a path, seeking to match traditional cardiovascular research values such as randomization and large population-based data samples with the individualized, targeted and patient-specific treatments and science in oncology. Future directions for the field will be carried partially by the application of broader interventional cardiology trends in cancer patients. Third-generation DES feature safer stent designs which could improve outcomes in patients with challenging coronary anatomies as well as biodegradable polymers. The advent of bioabsorbable vascular scaffold is a highly impressive innovation with unclear clinical value to date but promising applications in the population of cancer survivors. As a field addressing the intricate intersection between the top two leading causes of death in the United States, the challenge in interventional cardio-oncology is real but the potential for growth and expansion is massive.

**Table 1:** Anticancer Therapies Associated With Vascular Side Effects.

**Chemotherapy Agents**	**Adverse Cardiovascular Effects**	**Possible Mechanism**
**Antimetabolites**		
5-Fluorouracil	Angina, vasospasm, MI, SC	Vasospasm
Capecitabine	Angina, vasospasm, MI, SC	Vasospasm
Gemcitabine	Angina, vasospasm, MI	Vasospasm
**Antimicrotubule agents**		
Paclitaxel	Angina, vasospasm, MI	Vasospasm
Vinblastine (16, 79)	Angina, MI	Endothelial injury
**Monoclonal antibody-based tyrosine kinase inhibitor**		
Bevacizumab	Angina, MI, SC	Endothelial injury
**Small molecule tyrosine kinase inhibitors**		
Sorafenib	Angina, vasospasm, MI	Vasospasm
Sunitinib	Angina, MI, SC	Unknown
**BCR-ABL targeted tyrosine-kinase inhibitors**		
Nilotinib	Angina, MI, progression of CAD, PAD	Unknown
Ponatinib	Angina, MI, progression of CAD	Unknown
**Hormone therapy**		
Aromatase inhibitors (anastrozole, letrozole, exemestane)	Angina, MI	Unknown
Gonadotropin-releasing hormone agonists (goserelin)	Angina, MI	Unknown
**Radiotherapy**	Angina, MI, progression of CAD, PAD	Endothelial injury

MI indicates myocardial infarction; SC, stress-induced cardiomyopathy; CAD, coronary artery disease; PAD, peripheral artery disease.

## Author Contributions

Each of the authors contributed to the writing and editing of this manuscript. The first two authors contributed equally to this manuscript and are co-first authors.

## Conflict of Interest

The authors declare that the research was conducted in the absence of any commercial or financial relationships that could be construed as a potential conflict of interest.
